# Commentary: Corporate Social Responsibility: Deep Roots, Flourishing Growth, Promising Future

**DOI:** 10.3389/fpsyg.2016.00129

**Published:** 2016-02-15

**Authors:** William C. Frederick

**Affiliations:** Katz Graduate School of BusinessPittsburgh, PA, USA

**Keywords:** CSR stages, philanthropy, citizenship, regulation, sustainability

**A commentary on**

**“Corporate social responsibility: deep roots, flourishing growth, promising future,” in *The Oxford Handbook of Corporate Social Responsibility, Chapter 23***

*by Frederick, W. C. (2008). eds A. Crane, A. Williams, D. Matten, J. Moon, and D. S. Siegel (New York, NY: Oxford University Press, Inc.), 522–531.*.

This commentary expands upon an article of mine: “Corporate Social Responsibility: Deep Roots, Flourishing Growth, Promising Future,” published in 2008. The main goals of my commentary are to describe two levels of Corporate Social Responsibility (CSR)—macro and micro—and to advocate the need to integrate the two levels into a holistic analysis of CSR (Frederick, 2008).

The concept of Corporate Social Responsibility emerged in the United States at mid-twentieth century, advocated by both academic scholars and corporate executives. In 1951, Frank Abrams, chairman of the board of directors of America's largest oil company, advocated a “harmonious balance among stockholders, employees, customers, and the public at large” which is the very core of CSR's meaning. In 1953, a business school dean, Howard Bowen, wrote *The Social Responsibilities of the Businessman*, which was the first book to capture and summarize the main ideas about CSR (Frederick, 2006).

From that early beginning, the idea of Corporate Social Responsibility evolved in a series of stages:

- **CSR**_1_ (1950–1960s) proposed that corporate managers should act voluntarily and philanthropically as public trustees and social stewards.
- **CSR**_2_ (1960–1970s) broadened that idea to embrace legally-required corporate responses to many social demands.
- **CSR**_3_ (1980–1990s) called on businesses to develop ethical corporate cultures to support a wide range of stakeholders and communities through social contracts.
- **CSR**_4_ (1990–2000s) urged corporations to become global citizens heeding and correcting business's worldwide negative impacts on human societies and the natural environment^1^.

It is important to see that these CSR ideas and their proposed actions were aimed primarily at top-level managers and members of the firm's board of directors. Board members set the firm's policies, and the executive managers were responsible for putting those policies into action. In other words, CSR began with a “macro” focus that emphasized broad firm-wide policies, thereby laying the responsibility for attaining CSR results directly on top-level managers and the overall strategies they adopted. This was certainly the case during the first two stages of CSR development—CSR_1_ and CSR_2_—and even well into the CSR_3_ era. This firm-wide “macro” approach was intended to dampen and counteract the increasing numbers of social protests, new government regulations, and corporate scandals that focused a bright light on corporate misdeeds and socially irresponsible actions during the 1960, 1970, and 1980s^2^.

Whereas “Macro-CSR” focuses on top-level corporate policies and strategies, the focus of “Micro-CSR” is on the actual effects and impacts of those policies on people both inside and outside the corporation. In other words, what does Macro-CSR actually accomplish for the firm's employees, suppliers, customers, and citizens both local and far away? Surprisingly, the research literature of CSR deals mainly with “Macro-CSR” and far less with “Micro-CSR” issues^3^.

To fill that gap, the articles in this collection explore the various dimensions and meanings of Micro-CorporateSocialResponsibility, drawing upon a range of multidisciplinary concepts and research from the fields of organizational behavior, human relations, and psychology. Macro-CSR policies clearly have an impact on “people”: individual employees along the entire supply-chain (workers' human rights, decent working conditions, adequate pay), while similar policies and programs underwrite housing, meals, childcare, and healthcare for needy families and individuals at the Micro-CSR “people” level^4^.

Now, a new CSR stage—**CSR**_5_: **Sustainability** (2000–2050)—began with the opening of the new millennium. This stage reaches far beyond just the business corporation and its stakeholders, involving also the worldwide responsibilities of governments, international, and community organizations, and citizens from around the entire globe. Literally, Earthly life as we know it is now threatened and endangered by global warming, climate changes, rising ocean levels, and unlivable environmental pollution. Is Earthly Life itself sustainable? What will it take to attain that goal? Will “macro” global policies protect people at the “micro” level? I believe that an integrated, holistic solution will be sought, and hopefully found, by a coalition of “policy-makers” and “people”^5^.

I invite and urge you to read the papers in this collection to discover how the “Policy to People” goal can be approached and eventually attained.

## Author contributions

The author confirms being the sole contributor of this work and approved it for publication.

### Conflict of interest statement

The author declares that the research was conducted in the absence of any commercial or financial relationships that could be construed as a potential conflict of interest.

## Footnotes

^1^Details of each CSR stage may be found in Crane et al., 524–528.

^2^For a list of the major CSR missteps by corporations in this period, see “Landmark CSR Episodes and Crises” in Frederick, *Corporation, Be Good!*, 311–312.

^3^But changes were in the CSR wind. The most important shift toward a more inclusive view was R. Edward Freeman's pioneering concept of “stakeholder” in *Strategic Management:* A *Stakeholder Approach* (Marshfield, Massachusetts: Pitman Publishing, 1984), and the subsequent research that applied that concept to a wide range of stakeholders affected by corporate strategies. However, Freeman's main focus in the mid-1980s, as indicated by the book's title, was on top-level *strategic management*, i.e., clearly, the “macro” dimension of CSR.

^4^Many other examples of Micro-CSR are described in Wayne Visser, Dirk Matten, Manfred Pohl, *Nick Tolhurst (Editors), The A to Z of Corporate Socil Responsibility : A Complete Reference Guide to Concepts, Codes and Organizations*. (Chichester, UK: John Wiley & Sons, Ltd., 2007). See, for example, entries on Empowerment (of marginalized citizens), Health and Safety (of workers), Human Security (of people and their communities), Indigenous People (land rights, dignity), Labor Issues (in the workplace), Poverty (local and global), etc.

^5^For a comprehensive account of sustainability and its management, see Zabihollah Rezaee, *Business Sustainability : Performance, Compliance, Accountability and Integrated Reporting* (Sheffield, UK: Greenleaf Publishing, Ltd., 2015). For an analysis and discussion of sustainability challenges and opportunities facing corporate managers, see Chapter 10 in William C. Frederick, *Natural Corporate Management : From the Big Bang to Wall Street*, Sheffield, UK: Greenleaf Publishing, Ltd., 2012.
